# The Contribution of the PB1-F2 Protein to the Fitness of Influenza A Viruses and its Recent Evolution in the 2009 Influenza A (H1N1) Pandemic Virus

**DOI:** 10.1371/currents.RRN1006

**Published:** 2009-08-28

**Authors:** Vladimir Trifonov, Vincent Racaniello, Raul Rabadan

**Affiliations:** Columbia University

## Abstract

The absence of a full-length PB1-F2 protein has been suggested as one possible determinant for the low pathogenicity of the 2009 Influenza A H1N1 pandemic strain. Since the PB1-F2 sequence of this strain has three stop codons and its ancestors encode a full-length protein, the stop codons must have appeared recently. This suggests that the PB1-F2 protein is not evolutionary and functionally important for the new virus. We investigate the role of this protein in the evolution of influenza A viruses, and in particular in relation to the history of the new strain. We show that its evolutionary history is comparable to other, non-translated, subsequences in the PB1 segment, suggesting that PB1-F2 does not contribute significantly to the fitness of the influenza A virus.

## Introduction

On June 11th, 2009, the Director General of the World Health Organization [1] declared the first influenza pandemic of the 21st century. More than 30,000 cases were confirmed in 74 countries with 145 confirmed deaths [2]. Most of these infections were mild, and recovery was complete within a few days. However the initial reports from Mexico and several estimates of the case fatality rate (CFR) at 0.4% suggested that the new pandemic could be as severe as the one from 1957  [3].

Several determinants of influenza virus pathogenicity have been identified [4]. One of the most interesting is a second protein, called PB1-F2, encoded in reading frame +1 of the PB1 gene [5] [6]. PB1-F2 binds to mitochondria, leading to a release of cytochrome c and induction of apoptosis in CD8 T-cells and alveolar macrophages [6] [7]. In mice, PB1-F2 increases the severity of primary viral and secondary bacterial infections [8] [9], and has been associated with the high pathogenicity of avian H5N1 and the 1918 pandemic strain [10].

**Figure:**
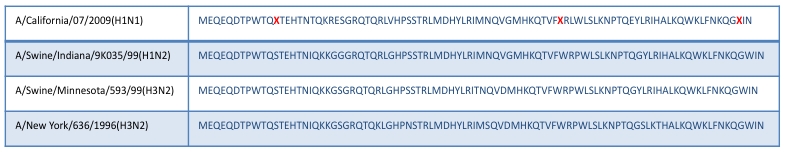
*Table 1.* *The PB1-F2 sequences of all ancestors of the 2009 Influenza A H1N1 strain do not contain stop-codons. The PB1-F2 sequence of the 2009 H1N1 strain contains three stop codons, which it has acquired in the last 10 years*

The PB1-F2 protein is truncated in the 2009 Influenza A (H1N1) strain, due to the presence of three stop codons at nucleotide positions 12, 58 and 88. The absence of a full-length PB1-F2 protein has been suggested to account for the low pathogenicity of the new pandemic strain [11] The PB1 segment of the recent strain is related to H1N2 and H3N2 swine viruses from 1998, and before that to human H3N2 viruses [12] [13] [14] *.* It is interesting to note that all the relatives of the new strain both in swine and human hosts encode a complete 90 amino acid long PB1-F2 protein (Table 1). The most likely scenario is that the complete PB1-F2 has been lost within the last ten years.

PB1-F2 appears truncated in classical swine H1N1 viruses and human H1N1 viruses since 1947. 96% of the avian viruses deposited in NCBI as of 2007 encode the complete (90 amino acid) version of the protein [15]. PB1-F2 has very high ratio of non-synonymous versus synonymous substitution, which can be explained by the higher conservation of PB1 and the weakly constrained protein sequence of PB1-F2 [16] [17] [18].

Because PB1-F2 appears truncated mainly in human and swine H1N1 viruses, one could speculate that in this specific evolutionary context PB1-F2 does not contribute to the fitness of the virus, and could be then truncated to a non-functional form. If this speculation was correct, the fact that the 2009 Influenza A (H1N1) acquired recently three stop codons in PB1-F2 leads to the conclusion that the PB1 segment of the pandemic strain has been circulating for the last few years in humans or swine hosts as part of H1N1 viruses. We argue that this reasoning is incorrect, although the conclusion could be correct. Our argument is based on the observation that independently of the influenza subtype there is no evidence for selective pressure to maintain full-length PB1-F2 in the viral genome compared with other subsequences within the same RNA segment that do not code for any protein.

## Methods

To study the evolution of the PB1-F2 sequence, we used the coding nucleotide sequences of the PB1 segment available in the NCBI database on June 14, 2009. For the analysis we used only full-length sequences that begin with a start codon and end with a stop codon. There were 2256 unique sequences with 1166 from avian hosts, 857 from human hosts, and 233 from swine hosts, which satisfied these criteria. Without the start and stop codon all of these sequences had a length of 2270 nucleotides. Since all pair-wise hamming distances were less than 0.21, we concluded that the sequences are aligned and did not perform any further alignment. We conducted three types of analyses on the sequences of the PB1 segment: the first to determine the distribution of stop codons in the six reading frames of the nucleotide sequences, the second to compare the evolutionary rate of PB1-F2 and other subsequences of PB1, and the third to estimate the probability of a random sequence to contain a long subsequence in reading frame +1, same as PB1-F2, without stop codons. 

**Figure:**
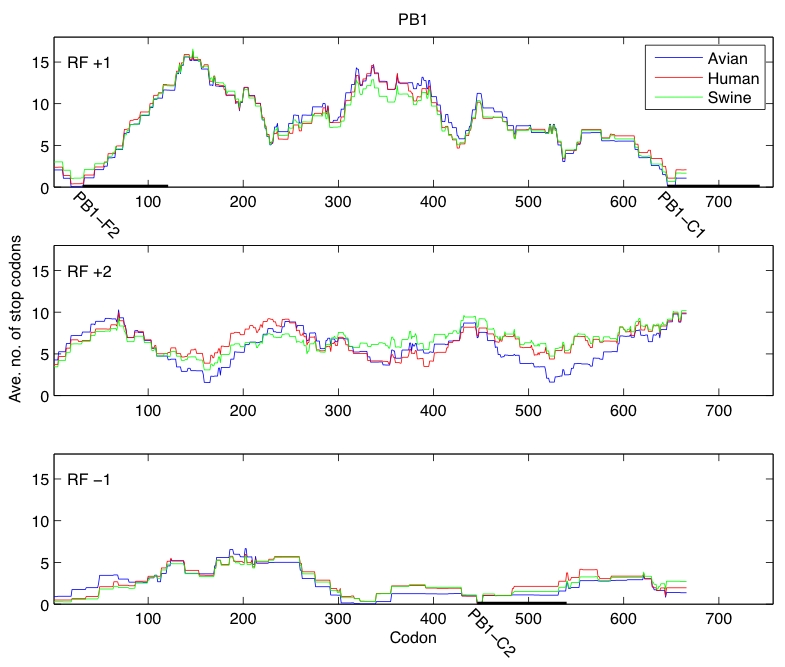
*Figure 1* *. Average number of stop codons in a window of length 90 for reading frames +1, +2, and -1 of the PB1 segment. Reading frame +1 contains PB1-F2 (codons 31-121) and PB1-C1 (codons 646-743). Reading frame -1 contains PB1-C2 (codons 446-540).*

In the first analysis, for each reading frame of the PB1 segment we considered the consecutive windows of length 90 codons and in each window determined the average number of stop codons present across all the PB1 sequences. We have shown the results of this analysis for reading frames +1, +2 and -1 in Figure 1 where the distribution of stop codons was plotted according to the host. In reading frame +1 the position of PB1-F2 is evident as a region with small number of stop codons close to the 5’ end of the PB1 segment (codons 31-121). Two more regions with a small number of stop codons are clearly distinguishable: one (PB1-C1) in reading frame +1 close to the 3’ of PB1 (codons 646-743) and one (PB1-C2) at codons 446-540 of reading frame -1.

**Figure:**
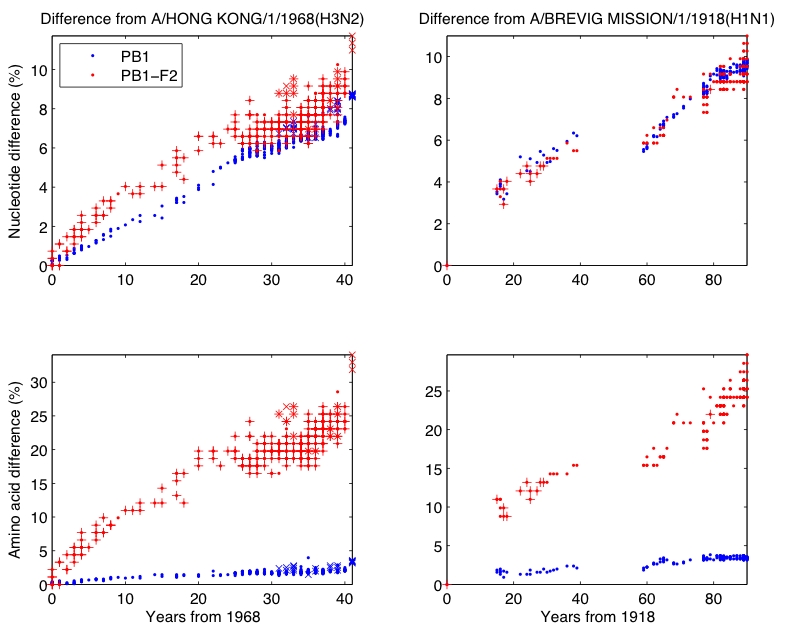
*Figure 2.* *Nucleotide/amino acid difference versus years difference of the influenza A strains from the 1968 and the 1918 pandemic lineage to the corresponding parent. Left two panels are for the 1968 pandemic lineage with parent A/Hong Kong/1/1968 (H3N2). The lineage contains human H3N2 (“.” mark), North American swine H1N2 (“x” mark), and 2009 human H1N1 (“x” mark). Right two panels are for the 1918 pandemic lineage with parent A/Brevig Mission/1/1918 (H1N1) containing human H1N1, except 2009 human H1N1. Top two panels are nucleotide difference, bottom two are amino acid difference. In each panel strains in which PB1-F2 is 90 amino acids long are marked with “+”.*

**Figure:**
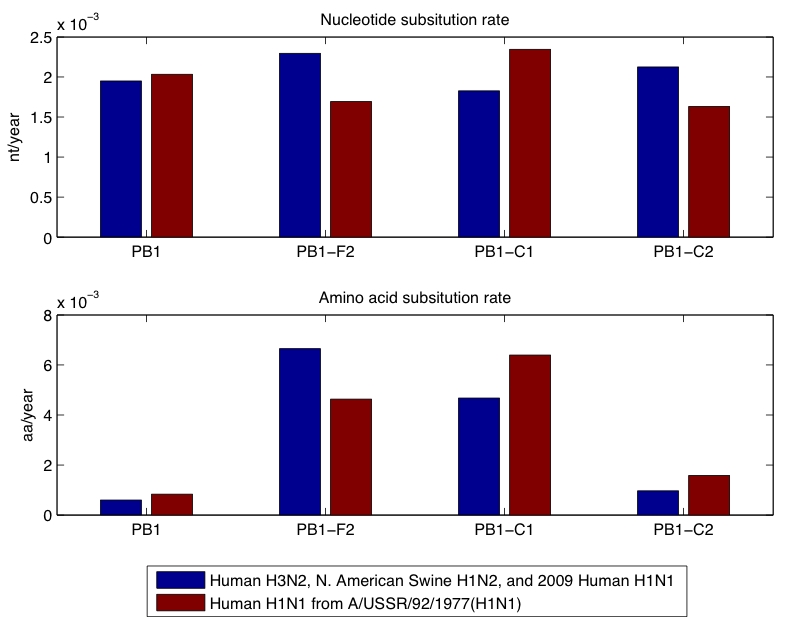
*Figure 3.* *Nucleotide and amino acid evolutionary rate of PB1, PB1-F2, PB1-C1, and PB1-C2 for the members of the 1968 and the 1918 pandemic lineage. The 1968 pandemic lineage contains human H3N2, North American swine H1N2, and 2009 human H1N1 with parent A/Hong Kong/1/1968 (H3N2). The 1918 pandemic lineage contains human H1N1 (except 2009 human H1N1) with parent A/USSR/92/1977 (H1N1).*

**

The second analysis was done separately on the PB1 sequences that have descended from the 1968 pandemic strain and the PB1 sequences that have descended from the 1918 pandemic strain. As descendants of the 1968 pandemic strain we used human H3N2, North American swine H1N2, and 2009 human H1N1 viruses. As descendants of the 1918 pandemic strain we used human H1N1, except 2009 human H1N1 because its PB1 segment descends through the North American swine H1N2/H3N2 from the 1968 pandemic strain. In Figure 2 we have shown the nucleotide and amino difference versus years difference of the members of each lineage to the corresponding parent of the lineage. Each plot contains separate data for PB1 and PB1-F2. In this figure as a parent of the 1968 pandemic lineage we took A/Hong Kong/1/1968 (H3N2) and for the 1918 pandemic lineage we took A/Brevig Mission/1/1918 (H1N1). The gap in the 1918 pandemic lineage between 1947 and 1977 is likely because this lineage was probably not circulating and non-evolving (“frozen”) [19]. Figure 3 shows the nucleotide and amino acid evolutionary rate of PB1, PB1-F2, PB1-C1, and PB1-C2 sequence in the two lineages. The evolutionary rate was determined as the coefficient in a least square fit of a linear model with zero constant term and with input and response variables correspondingly the years difference and the nucleotide/amino acid difference of a subsequence in a lineage to the parent of the lineage. To avoid problems with the 1947-1977 gap of the 1918 pandemic lineage we took only the part of this lineage after 1977 and took A/USSR/92/1977 (H1N1) as a parent of the lineage.

**Figure:**
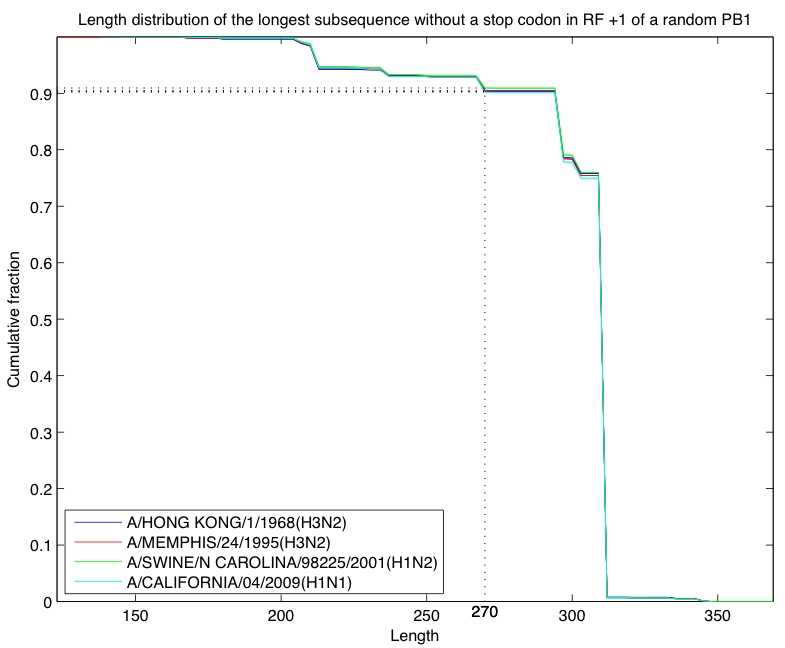

***Figure 4.*** *Length distribution of the longest subsequence without a stop codon in reading frame +1 of a random PB1 sequence.* *The random sequence was selected according to the model described in the text and probabilities were estimated by sampling 10,000 random sequences.* *As a starting sequence we used four sequences from the H3N2 lineage descending from the 1968 pandemic strain. In all sequences except A/California/04/2009 (H1N1) the PB1-F2 segment does not contain stop codons. In all four cases the probability of length at least 270 is close to 0.91.*

For the third analysis we defined a simple model of a random nucleotide sequence. The model is parameterized by an amino acid sequence (the starting sequence) and a codon bias given for every position of that sequence. The model generates a random nucleotide sequence of length three times the length of the starting amino acid sequence. Each codon of the random sequence is picked at random among the codons that translate to the amino acid located at that position in the starting sequence and using the codon bias for that position. Note that the random nucleotide sequence translates to the starting amino acid sequence. We are interested in the distribution according to this model of the length of the longest subsequence without stop codons in reading frame +1 of the random sequence. As a starting sequence we took the PB1 sequence of four strains from the H3N2 lineage descending from the 1968 pandemic strain. In all four cases the codon bias for each position was fixed to be the codon bias calculated for that position across all the 2256 coding sequences of the PB1 segment described at the beginning of this section and the length distribution was estimated by generating 10,000 random sequences (Figure 4). In all cases the probability of having a subsequence in reading frame +1 of length at least 270 and without stop codons was estimated to be close to 0.91.

## Results and Discussion

Since the appearance of the H1N1 pandemic strain in March 2009 there has been an intense effort to evaluate possible factors that could contribute to its pathogenicity. One of these factors is a small protein, called PB1-F2, encoded in the PB1 segment of previous pandemic strains. This protein has been shown to cause apoptosis of immune cells, contributing to increased viral virulence and secondary infections. Fortunately, the PB1-F2 protein is truncated in the 2009 H1N1 pandemic strain, although the swine and human ancestors of the virus encode a full-length protein. 

Because the full-length PB1-F2 protein is absent from many influenza viruses, its evolutionary role and contribution to the fitness of the virus is unclear. An important question is whether the protein is necessary for virus survival in different animal populations. The PB1-F2 protein is truncated in many human and swine viruses. Our approach to answering these questions is to compare the evolution of PB1-F2 with PB1 and two other “control” open reading frames within the same segment, PB1-C1 and PB1-C2, which do not appear to encode any proteins. Both genomic sequences have open reading frames of length comparable to PB1-F2 and are as conserved as PB1-F2. PB1-C1 is 98 amino acids long and in the same reading frame as PB1-F2. It is unlikely that it encodes any functional protein as the start codon is at the end of the PB1 segment. PB1-C2 is a 97 amino acid sequence in the negative strand, the same as the viral RNA. 

While PB1 does have a low evolutionary rate at amino acid level (Figure 3), PB1-F2 does not display any particular sign of negative selection. In the descendants of the 1977 H1N1 human viruses, PB1-F2 is truncated while it is complete in the H3N2 human viruses. Amino acid evolutionary rates in the truncated and complete versions are similar. Even more relevant is the comparison with the two control segments. PB1, PB1-F2 and the two control segments show similar evolutionary rates at the nucleotide level but very different rates at the amino acid level. Both PB1 and PB1-C2 show lower rates of evolution at the amino acid level than PB1-F2 and PB1-C1. For PB1 the lower evolutionary rate is probably a consequence of negative selection and for PB1-C2 the lower evolutionary rate can be explained by the fact that the third codon positions in reading frame -1 coincide with third codon positions of PB1 itself. The higher rates in PB1-F2 and PB1-C1 can be explained by the frame shift relative to PB1, so that synonymous mutations in PB1 appear as non-synonymous in those sequences.  In summary, the amino acid evolutionary rates in all sequences can be explained entirely by negative selection in PB1. This fact coincides with previous observations (6) that show that the higher dn/ds ratio of PB1-F2 is due to negative selection in the PB1 protein.

PB1-F2 is complete in all H1N1 human isolates before 1947, at which time a stop codon appeared which truncated the protein to a shorter (57 amino acid version). If the longer version of the protein conferred a functional advantage, one would expect a change in the evolutionary rates of the human H1N1 PB1-F2 proteins in 1947. This change is not observed (Figure 2), and the rates before and after are similar to evolutionary rates in the human H3N2 viruses that encode a complete version of the protein.

Even though PB1-F2 is not particularly conserved, one could still consider notable the fact that it is present as a long open reading frame in most influenza isolates. The importance of this observation is diminished by the fact that it also applies to both PB1-C1 and PB1-C2 and furthermore holds for a PB1 segment generated at random. Figure 2 shows that the average number of stop codons in PB1-F2 is low, but not lower that any of the control sequences. Figure 4 shows that the probability of a long subsequence without stop codons in the reading frame of PB1-F2 of a PB1 segment generated at random is high (>0.9). The coding regions for PB1-C1 and PB1-C2 have an even lower number of stop codons in all the influenza isolates that have been deposited at NCBI. The observation that PB1-F2 is truncated in some of the human and swine strains also applies to the other control sequences. This analysis, as the previous ones, suggest that PB1-F2 has a similar contribution to the fitness of the virus as other non-translated sequences, PB1-C1 and PB1-C2, which suggests that PB1-F2 is of little or no evolutionary significance for the virus.

In summary, the PB1-F2 open reading frame appears to be as conserved, and maintained as a full-length protein, as other non-coding regions of the same RNA segment and of a PB1 segment generated at random. These observations, and the fact that PB1-F2 is truncated in many virus isolates, suggest that the evolutionary role of PB1-F2 in vivo is minimal. Although PB1-F2 clearly effects viral replication and virulence in mice, more extensive studies are required to assess the contribution of the PB1-F2 protein to the fitness of the influenza virus.

Funding information

The authors have no support or funding to report.

Competing interests

The authors have declared that no competing interests exist.

## References

[ref-3505042616] “Pandemic (H1N1) 2009” on WHO website. http://www.who.int/csr/disease/swineflu/en/.

[ref-1729062109] “World now at the start of the 2009 influenza” on WHO website. http://www.who.int/mediacentre/news/statements/2009/h1n1_pandemic_phase6_20090611/en/.

[ref-4040976329] C. Fraser et al., Science 324, 1557 – 1561 (2009). 10.1126/science.1176062 PMC3735127 19433588

[ref-280878417] G. Neumann, T. Noda, Y. Kawaoka, Nature 459, 931 – 939 (2009). 10.1038/nature08157 PMC2873852 19525932

[ref-876958108] W. Chen et al., Nature Med. 7, 1306–1312 (2001). 10.1038/nm1201-1306 11726970

[ref-2832711885] G. M. Conenello, P. Palese, Cell Host & Microbe 2, 207 – 209 (2007). 10.1016/j.chom.2007.09.010 18005736

[ref-1686354999] D. Zamarin, A. Garcia-Sastre, X. Xiao, R. Wang, P. Palese, PLoS Pathog. 1, e4. doi:10.1371/journal.ppat.0010004. 10.1371/journal.ppat.0010004 PMC1238739 16201016

[ref-3246371002] D. Zamarin, M. B. Ortigoza, P. Palese, J. Virol. 80, 7976 -- 7983 (2006). 10.1128/JVI.00415-06 PMC1563817 16873254

[ref-2140133537] J. L. McAuley et al., Cell Host & Microbe 2, 240 – 249 (2007). 10.1016/j.chom.2007.09.001 PMC2083255 18005742

[ref-4030844575] G. M. Conenello, D. Zamarin, L. A. Perrone, T. Tumpey, P. Palese, PLoS Pathog. 3, e141. doi:10.1371/journal.ppat.0030141. 10.1371/journal.ppat.0030141 PMC2000966 17922571

[ref-1791466934] T. Taia, R. Wang, P. Palese, Cell 137, 983 – 985 (2009). 10.1016/j.cell.2009.05.032 19524497

[ref-2285027613] V. Trifonov, H. Khiabanian, R. Rabadan, NEJM 360, 115 – 119 (2009). 10.1056/NEJMp0904572 19474418

[ref-2578196736] A. Solovyov, G. Palacios, T. Briese, W. I. Lipkin, R. Rabadan, Euro Surveill. 14, 2009. 10.2807/ese.14.21.19224-en PMC4310691 19480812

[ref-2169696406] V. Trifonov, H. Khiabanian, B. Greenbaum, R. Rabadan, Euro Surveill. 14, 2009. 19422769

[ref-835895729] R. Zell et al., J. Gen. Virol. 88, 536 – 546 (2007). 10.1099/vir.0.82378-0 17251572

[ref-42724037] J. C. Obenauer et al., Science 311, 1576 – 1580 (2006). 10.1126/science.1121586 16439620

[ref-1228994987] J. C. Obenauer, Y. Fan, C. W. Naeve, Science 313, 1573 (2006).

[ref-1287696265] E. C. Holmes, D. J. Lipman, D. Zamarin, J. W. Yewdell, Science 313, 1573 (2006). 10.1126/science.1131729 16973862

[ref-2969220631] K. Nakajima, U. Desselberger, P. Palese, Nature 274, 334 – 339 (1978). 10.1038/274334a0 672956

